# Valve-Like and Protruding Calcified Intimal Flap Complicating Common Iliac Arteries Kissing Stenting

**DOI:** 10.1155/2015/451962

**Published:** 2015-12-09

**Authors:** George S. Georgiadis, Efstratios I. Georgakarakos, Nikolaos Schoretsanitis, Christos C. Argyriou, George A. Antoniou, Miltos K. Lazarides

**Affiliations:** ^1^Department of Vascular Surgery, “Democritus” University of Thrace, University General Hospital of Alexandroupolis, Greece; ^2^Liverpool Vascular and Endovascular Service, Royal Liverpool University Hospital, Liverpool, UK

## Abstract

Endovascular therapy for iliac artery chronic total occlusions is nowadays associated with low rates of procedure-related complications and improved clinical outcomes, and it is predominantly used as first-line therapy prior to aortobifemoral bypass grafting. Herein, we describe the case of a patient presenting with an ischemic left foot digit ulcer and suffering complex aortoiliac lesions, who received common iliac arteries kissing stents, illustrating at final antegrade and retrograde angiograms the early recognition of a blood flow obstructing valve-like calcified intimal flap protruding through the stent struts, which was obstructing antegrade but not retrograde unilateral iliac arterial axis blood flow. The problem was resolved by reconstructing the aortic bifurcation at a more proximal level. Completion angiogram verified normal patency of aorta and iliac vessels. Additionally, a severe left femoral bifurcation stenosis was also corrected by endarterectomy-arterioplasty with a bovine patch. Postintervention ankle brachial pressure indices were significantly improved. At the 6-month and 2-year follow-up, normal peripheral pulses were still reported without intermittent claudication suggesting the durability of the procedure. Through stent-protruding calcified intimal flap, is a very rare, but existing source of antegrade blood flow obstruction after common iliac arteries kissing stents.

## 1. Introduction

Aortobifemoral bypass is still considered the gold standard treatment for chronic total occlusions (CTOs) of iliac arteries that belong to Transatlantic Intersociety Consensus (TASC II) guidelines C/D lesions [1]. This procedure reportedly results in 80–90% patency at 5 and 10 years, although according to a meta-analysis it is associated with an aggregated operative mortality rate of 4.4% and a major-complication rate of 12.1% [2]. However, endovascular therapy for TASC II C/D lesions has also evolved during the past 2 decades [1, 3, 4] offering less invasive treatment and shorter hospital stays, and, notably, treatment of TASC D lesions is increasingly adopted as a first-line management option, not only for patients considered unfit enough for open surgical treatment [3–5]. Recent studies using bare metal stents in the common iliac arteries (single or bilateral with an overlap in the distal aorta) for the treatment of bilateral aortoiliac occlusive disease have demonstrated a high rate of technical success, good mid- and long-term patency, and low procedural morbidity and mortality [3, 4, 6–8]. This is more prominent when primary stenting is performed instead of transluminal angioplasty followed by selective stenting [9]. Reported results for technical success and long-term patency are only slightly poorer than those obtained in the treatment of stenosis [10].

Occasionally, procedure-related complications may occur in both single- or kissing stent technique, necessitating various troubleshooting techniques or bailout procedures [3]. We describe herein the case of a patient presenting with an ischemic left foot digit ulcer and suffering left common iliac artery (CIA) CTO and contralateral CIA severe stenosis, who received CIA kissing stents. The final, antegrade and retrograde angiograms revealed a blood flow obstructing valve-like calcified intimal flap that was protruding through the stent struts and obstructing the antegrade, but not the retrograde left iliac axis blood flow, and necessitating the reconstruction of the aortic bifurcation at a more proximal level.

## 2. Case Presentation

**Figure 1:**
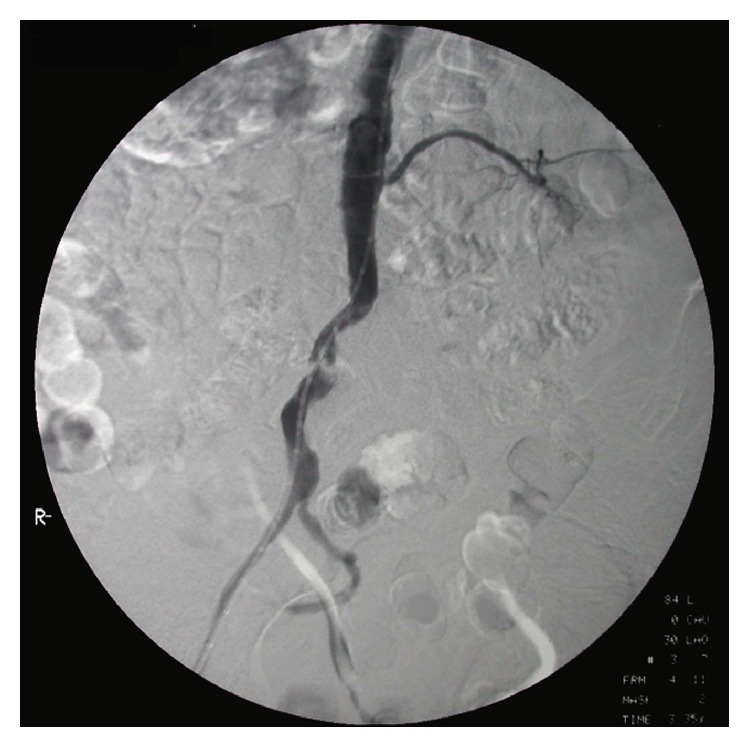
Patient's preoperative intra-arterial subtraction angiography revealing TASC D aortoiliac lesions.

A 79-year-old man presented with deterioration of left lower extremity critical ischemia, including the appearance of a small ischemic ulcer on the first digit. He was a chronic heavy smoker and had chronic obstructive pulmonary disease. Furthermore, he was obese (BMI > 30 kg/m^2^). A clinical examination demonstrated an absent ipsilateral and a weak contralateral femoral pulse. His ankle brachial indexes were 0.32 and 0.64, respectively. Digital subtraction angiography revealed CTO of the left iliac axis and common femoral artery, and severe (~80%) right CIA stenosis (Figure 1).

Considering his severe comorbidities, the patient was scheduled for an endovascular approach. A* Terumo* hydrophilic 0.035′′ 180-cm guide wire (Terumo Company, Tokyo, Japan) was placed percutaneously through the right femoral artery and advanced into the aorta. A similar, but through open femoral cut-down, guide wire was placed within the occluded left iliac axis, supported by a 5 × 6 cm* Optimed* (OptiMed, Ettlingen, Germany) percutaneous transluminal angioplasty dilatation catheter. This eventually crossed the lesion intraluminally, and was then advanced into the aorta. Balloon angioplasty was performed initially to predilate iliac arteries lesions before self-expandable stents (SEs) were implanted. Subsequently, primary CIA stenting was performed using standard techniques with a 10 × 40 mm* Complete* SEs (Medtronic Santa Rosa, CA, USA) on the right and a 10 × 100 mm* E-Luminexx* SEs (Bard Peripheral Vascular, Tempe, AZ, USA) on the left side, raising the aortic blood flow divider by 2-3 mm. After stent deployment, concomitant postdilatation was performed using the kissing balloon technique. At a later stage, the left-sided SEs were extended up to the distal end of external iliac artery with a new* E-Luminexx* SEs (7 × 60 mm), to fully cover the occluded lesion and completely recanalize the left iliac artery axis.

**Figure 2:**
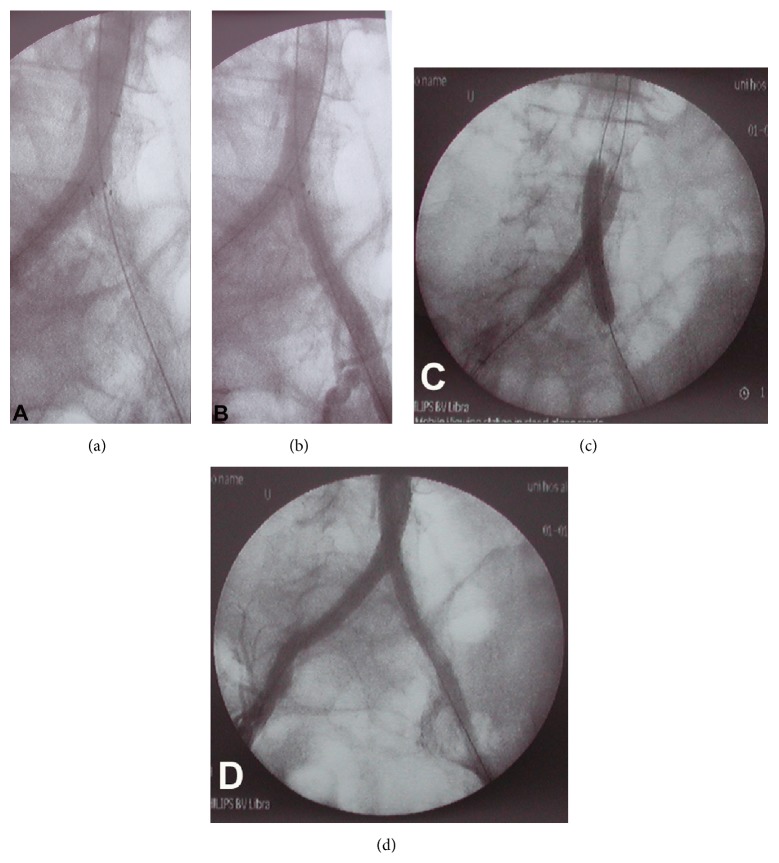
(a) Antegrade angiogram shows obstruction of left iliac artery axis blood flow, from an elevated valve-like calcified intimal flap protruding through the upper pole struts of the left-sided kissing stent. (b) Retrograde contrast injection reduces the obstructing flow flap against the stent wall, demonstrating normal aortoiliac anatomy. (c) Raising the aortic bifurcation with another kissing technique entirely traps the calcified flap between the two stents. (d) Angiogram demonstrating normal aortoiliac blood flow.

**Figure 3:**
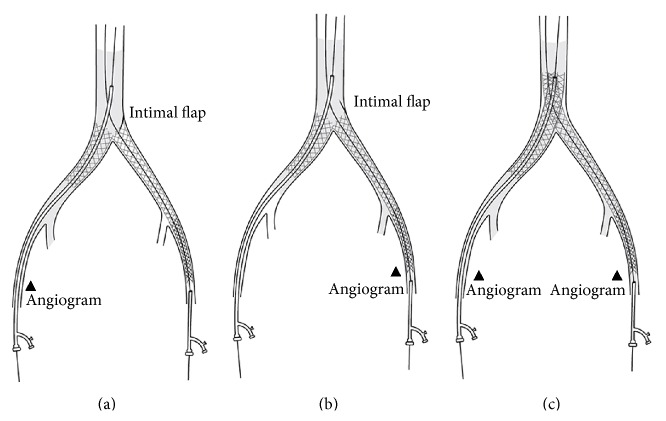
Schematic representation of the steps of the procedure and bailout kissing stenting.

Completion angiograms were obtained postoperatively on both sides, but not simultaneously. Obstruction of left iliac axis antegrade flow was revealed after injecting contrast agent through a right-side-inserted* Arrow* catheter (Arrow International Inc., USA) placed into the aorta, 1-2 cm above the kissing stents (Figures 2(a) and 3(a)). However, contrast agent injected retrograde through a short 7 Fr × 11 cm sheath placed through the left femoral artery revealed normal aortoiliac visualization (Figures 2(b) and 3(b)).

An intimal calcified flap protruding through the struts of the upper pole of the left-sided SEs was evident in the obtained images. Revascularization was achieved by raising the aortic bifurcation (~2 cm) using new kissing balloon expandable stents (BEs, 9 × 60 mm,* Assurant*) (Medtronic, Santa Rosa, CA, USA) inside the SEs (telescope technique), thus trapping the calcified intimal plaque. New angiograms (antegrade and retrograde) obtained after concomitant expansion of the stents verified normal aortoiliac blood flow (Figures 2(c), 2(d), and 3(c)). Normal proximal femoral pulses were detected after completing the procedure. Additionally, a severe left femoral bifurcation stenosis was also corrected by endarterectomy-arterioplasty with a bovine patch. Postintervention ankle brachial pressure indices were significantly improved. Postoperatively, long-term monotherapy with clopidogrel (75 mg/day) was applied. The wound ulcer healed within two weeks of revascularization. At the 6-month and 2-year follow-up, normal peripheral pulses were still reported without intermittent claudication suggesting the durability of the procedure.

## 3. Discussion

Recanalization of heavily calcified and occluded iliac vessels (TASC I D lesions) can be challenging. Treatment of iliac CTOs may initially be limited primarily by an inability to cross the occlusion and later by the failure of balloon angioplasty [11]. However, the development of stents to treat failed angioplasty led to the inability to cross back into the true lumen after crossing the occluded segment, becoming the primary cause for acute procedural failure in the treatment of iliac CTOs. Total unilateral iliac arterial axis occlusion can be approached in a similar way, as described by Palmaz et al. [12], with omission of the contralateral stent especially if the contralateral CIA ostium has no disease or has an intermediate stenosis [3]. However, in asymmetric aortoiliac lesions the application of large displacement forces to severely calcified and irregularly shaped plaques during unilateral iliac artery dilatation may augment contralateral iliac occlusion due to plaque shifting and embolization to the contralateral iliac artery. For this reason, we preferably use the kissing stent technique to overcome such complex, calcified, and eccentric plaques even if patients suffer predominantly from unilateral arterial pathology. Two simultaneously deployed BEs or two simultaneously expanded SEs are implanted, one in each CIA, protruding into the distal aorta and making parallel contact. These two stents effectively exclude atherosclerotic aortic bifurcation plaques from the circulation, trapping them between the struts of the stent and the arterial wall. Conventional kissing stenting which allows full coverage of bifurcated lesions has been reported also in other arterial lesions [13].

Currently, there is no consensus whether the kissing stents technique offers an advantage over the single-stent stenting strategy [3, 4]. Furthermore, studies showed that stent type or stent configuration (single, V or kissing) is not a predictor of primary patency in true bifurcation lesions [4, 14]. Most authors have reported favorable results after reconstructing the aortoiliac bifurcation with the kissing stent technique, with technical success rates between 89% and 100% [3, 4, 8] and with primary patency rates between 76% and 98% at 1 year [3, 4, 8, 15] and between 77.6% and 87% during a follow-up of 30–36 months [3, 14, 15]. Secondary patency rates were estimated to be 98.2% at 1 year [4] and between 97.5% and 98% at 36 months [3, 15]. Furthermore, there are numerous significant factors related to outcome. Prior data suggest that age >50 years and patients without an iliac occlusion were independent predictors of comparable to surgery long-term stent patency rates [16]. In another study, female gender and diameter stenosis after endovascular therapy but not CTO, TASC II C/D lesions, stent configuration, or stent type were reported as factors influencing primary patency in bifurcation lesions [4]. Overall, heterogeneity in study populations, lesion characteristics, and applied procedural techniques may explain the variations in immediate and late outcomes.

TASC II C/D lesions are generally regarded as challenging targets to treat due to their high plaque burden and their anatomical proximity of the aorta and both CIAs. Occlusion of a contralateral CIA due to unfavorable plaque shift has a low incidence using the single-stent technique [3]. However, in bilateral implantation of stents, plaque shift and atherosclerotic progression into one of the stents necessitating bailout new stenting is a very rare event. Furthermore, in our case the calcified arterial plaque protruding through the struts of the left CIA SEs resulted in the obstruction of the antegrade blood flow, but it permitted the retrograde flow when the angiogram was performed from the ipsilateral femoral artery short sheath. This fact suggests that, even covering disease with stents, from healthy to healthy areas, some intimal disease will always be present in atherosclerosis [17]. It is possible that some highly calcified lesions overcome the radial force of the bare metal stents especially in small diameters and protrude even within the stent struts.

It has been shown also that patients in whom the proximal end of the kissing stents overlapped more than half of their angiographic width within the aorta had significantly lower primary and assisted primary patency rates at 2 years, compared to those in whom the proximal ends of the stents overlapped half of their width or less [18]. In our case, the overlap of the free proximal stent ends in the distal aorta was only a few millimetres, suggesting that other reason leaded to the initial failure of kissing stents in the aortic bifurcation and was not an application error. Although this problem was treated easily by raising the aortic bifurcation with another two kissing stents, this case highlights the importance of obtaining both antegrade and retrograde angiograms in order to identify such flow-obstructing valve-like intimal flaps. Traditionally, completion angiography through a single flush catheter placed at one femoral side is sufficient to visualize the aortoiliac arterial segment after aortoiliac kissing stenting. The presence of left CIA SEs thrombosis would be assumed if this procedure is performed from the right side and is combined with a lack of contralateral femoral pulses, necessitating acute thrombolysis or possible conversion to femoral-femoral crossover bypass. It is therefore crucial to recognize this pitfall early and subsequently correct it with an appropriate procedure.

The potential shortfall of retrograde completion angiography and the contrasting benefit of antegrade angiography in depicting neointimal flaps were shown recently in aortobifemoral grafts [19]. As in our case, the novel utilization of a standard endovascular method (raising the graft bifurcation) corrected flaps that involved the graft body. However, the authors of that report raised concerns regarding retrograde injections of contrast agent within the limbs of aortobifemoral grafts, since hemodynamic forces from a distal to a proximal level might otherwise reduce any flaps against the graft wall. This phenomenon occurred in our case with the calcified flap being fully adherent to the arterial wall when retrograde injection was applied but separated from it when antegrade injection was performed, facilitating its definition radiographically. The possibility that the initial stent was through a dissection caused by recanalization and subsequently corrected is not supported since all attempts occurred intraluminally without any recognition of a dissecting plane or a dissecting flap on retrograde angiograms prior to eventual success. We recognize also that intraoperative computed tomography and intravascular ultrasound are the only presently available methods to objectively demonstrate the intimal flap protruding through the struts. However, the first method is not widely available, although it increases operation time and radiation exposure, while intravascular ultrasound, a rather more convenient method at least in the United States, is not available in most European vascular surgery units (and in our department). Additionally, cumulative experience is needed to be used with safety and without prolonging the operative time.

Novel minimally invasive techniques such as the single-stent procedure eventually result in the tacking of intimal calcified flaps or calcified plaques that protrude through prior stents and allow for normal antegrade blood flow without hemodynamic compromise. However, ostia CIA flaps require kissing stents to compress them between the stent and the arterial wall, simultaneously protecting the contralateral side. Similar stent-in-stent techniques have been used to treat plaque prolapse in stents placed in the coronary circulation [20]. Although multiple in-stent techniques may increase the metal surface area, which might in turn cause subacute stent thrombosis or restenosis, the large valve-like calcified flap in the present case caused severe blood flow limitation (a preocclusive lesion) in the left iliac artery, which could result in subsequent abrupt closure or subacute stent thrombosis. The additional kissing stenting was therefore performed, which successfully excluded the flap from the circulation. Covered stents are also able to exclude plaque and endothelium and their use might be beneficial in our case, but they are more expensive than bare metal stents. Although covered and bare metal balloon expandable stents produce similar and acceptable results for TASC B lesions, their better performance for TASC C and D lesions regarding longer-term patency and clinical outcome should not be overlooked [18, 21].

## 4. Conclusion

Through bare metal stent-protruding heavily calcified intimal flap overcoming the radial force of the stent is a very rare but existing source of antegrade blood flow obstruction after CIA's kissing stents for aortoiliac occlusive disease. Interventionalists should remember this procedural complication and all effective ways to treat it endovascularly when treating complex aortic bifurcation lesions. As such, no clinical consequences for the patient will arise making the procedure extremely safe.
